# Synthesis of a Novel Siliconized Analog of Clofibrate (Silafibrate) and Comparison of their Anti-inflammatory Activities

**Published:** 2012

**Authors:** Mojtaba Ziaee, Morteza Samini, Mohammad Bolourtchian, Mohammad Ghaffarzadeh, Maryam Ahmadi, Mohammad Ali Egbal, Arash Khorrami, Sina Andalib, Nasrin Maleki-Dizaji, Alireza Garjani

**Affiliations:** a *Department of Pharmacology, Science & Research Branch, Islamic Azad University, Tehran, Iran.*; b *Chemistry and Chemical Engineering Research Center of Iran, Tehran, Iran.*; c *Department of Pharmacology & Toxicology, Faculty of Pharmacy, Tabriz University of Medical Sciences, Tabriz, Iran. *

**Keywords:** Clofibrate, Silicon, Siliconized analog, Anti-inflammatory

## Abstract

Fibrates, as hypolipidemic drugs known as agonists of peroxisome proliferator-activated receptors, diminish inflammatory responses. Studies have shown that incorporation of a silicon atom into a drug structure improves its pharmacological potency, modifies its selectivity toward a given target, or changes its metabolic rate, in addition to increasing the lipophilicity of the compounds. A siliconized analog of clofibrate, ethyl-2-methyl-2-(4-(trimethylsilyl)phenoxy)propionate was synthesized, whereby the chlorine atom in the phenoxy ring was replaced by a trimethylsilyl group. The anti-inflammatory effects of the siliconized analog (silafibrate) were evaluated in an air-pouch model of inflammation and compared with those of clofibrate. Oral administration of both drugs produced a significant anti-inflammatory action by reducing carrageenan induced pouch leukocyte recruitment, exudates production, and granulated tissue weight. The silicon isostere of clofibrate has improved anti-inflammatory properties.

## Introduction

Clofibrate is a halogenated fibric acid derivative that is used in the treatment of hypertriglyceridemia. This effect is mediated through its interaction with peroxisome proliferator-activated receptor (PPAR)-α receptors, which results in the stimulation of fatty acid oxidation, increased lipoprotein lipase synthesis, and reduced expression of apolipoprotein C-III (1). Fibrates differ in their PPAR-*α* binding potency, and a structure–activity relationship appears to exist (2). Bezafibrate, ciprofibrate, and fenofibrate are halogenated molecules, with a phenoxy-2-methyl-2-propanoic acid chain, and their structures are similar to clofibrate as a parent analog (3). Increasing data show that activation of PPAR-*α* receptors also suppresses inflammation (4); therefore, fibrates possess anti-inflammatory properties (5). 

The discovery of the biological target of fibrates-PPARs, specifically the alpha isotype, enabled the explanation of the diverse lipid-lowering and non-lipid effects of fibrates, which contribute to their hypolipidemic and antiatherosclerotic benefits (6, 7). Synthesis of isosteres is one of the methods by which new putative drugs are found based on the structures of existing drugs (8, 9). Silicon is an element similar to carbon, because it forms four covalent bonds with many other elements (10). Silicon-based drugs are now being developed, and silicon-containing compounds have entered human clinical trials. Siliconized compounds used as medical agents have been shown to retain or improve their biological profiles in comparison with those of their corresponding isosteres (11). 

In this study, we synthesized a siliconized derivative of clofibrate, named silafibrate, by replacing the chlorine atom in the phenoxy ring with a trimethylsilyl group (Figure 1) and compared its inhibitory effect on leukocyte accumulation with that of clofibrate in a carrageenan-induced air-pouch model of inflammation in rats.

## Experimental

*Chemicals*

A siliconized analog of clofibrate, silafibrate, was synthesized in the Chemistry and Chemical Engineering Research Center, Tehran, Iran. Clofibrate was gifted by Zahravi Pharmaceutical Inc (Tabriz, Iran); penicillin-G potassium and dihydrostreptomycin sulfate were gifted by Daana pharm (Tabriz, Iran). Carrageenan was obtained from Sigma Company (Germany). All other chemicals were of the highest grade available commercially.

*Synthesis of p-trimethylsilylphenol*

*p-*Trimethylsilylphenol was synthesized with slight modifications of an earlier method reported by John L. Speier (11). The route of synthesis *p-*Trimethylsilylphenol is outlined in Figure 2.

Step 1: Dry *p-*chlorophenol (25.7 g, 0.2 moles) was mixed with an excess amount of trimethylchlorosilane (33.6 g, 0.31 moles) in a flask equipped with a condenser and was heated to reflux. A vigorous flow of hydrogen chloride gas was produced. After 6 hours, the reaction mixture was distilled to recover the unreacted excess of trimethylchlorosilane. Chlorophenoxy-trimethylsilane was obtained quantitatively. 

Step 2: Sodium (10 g, 0.43 moles) was melted in hot toluene (40 mL) in a flask equipped with a condenser and a dropping funnel. Trimethylchlorosilane was added to the molten sodium using the dropping funnel to decrease the boiling point of the mixture (to about 101°C). The process continued vigorously.* p-*Chlorophenoxytrimethylsilane (40 g, 0.2 moles), the only product of the previous step, was mixed with the residue of trimethylchlorosilane and further added extremely slowly to sodium through the dropping funnel, with simultaneous stirring of the mixture, under a reflux condenser. When the addition was complete, the reaction mixture was stirred and heated for half an hour and allowed to be stirred overnight (with no more heating).

*P-Trimethylsilylphenoxy-trimethylsilane* was obtained by distillation of the product mixture.

Step 3: Trimethylsilylphenoxytrimethylsilane (24 g, 0.1 moles) was dissolved in 15 ml of 95% ethyl alcohol. It was acidified with just one drop of 25% hydrochloric acid and diluted with 3 ml of water while stirring. The mixture started to become warm immediately. The reaction vessel was placed in an ice-bath and chilled; afterward, it was diluted with 6 mL water while stirring. The turbidity of the mixture increased continuously. Then, it was maintained undisturbed for 15 min. The organic layer was separated and placed in a large crystallizer plate. After crystallization, white needles of *p-*Trimethylsilylphenol were obtained (m.p.: 74–74.2 °C).

^1^H-NMR (CDCl_3_) ^TM^ 7.45 (d, J = 8.5 Hz, 2 H), 6.89 (d, J = 8.5 Hz, 2 H), 4.98 (b, 0 H), 0.29 (s, 9 H)

*Synthesis of silafibrate*

*Preparation of silafibric acid*

Chloroform (4 g) was added drop-by-drop to a flask containing* p-*trimethylsilyl-phenol (5 g), potassium hydroxide (1.68 g), and acetone (100 mL); the reaction mixture was refluxed for 4–5 hours. The excess of acetone was distilled, and the reaction mixture was dissolved in water, decolorized with charcoal, and then acidified with diluted (25%) HCl. After extraction with dichloromethane and washing of the organic phase thus obtained with saturated NaHCO_3_ solution, it was acidified with HCl solution; thus, the pure product was obtained.

*Esterification of silafibric acid*

In a flask containing *p-*trimethylsilyl-phenol (3 g), equipped with a Dean-Stark apparatus, ethanol (50 mL) and toluene (70 mL) were added and refluxed overnight, trapping the water produced in the Dean-Stark receiver. The mixture was subjected to rotary evaporation under vacuum, and the resulting oily residue was dissolved in dichloromethane and washed with a solution of saturated NaHCO_3_ solution. The organic phase was dried over anhydrous sodium sulfate and stripped from the solvent under vacuum to produce pure silafibrate. The route of synthesis ethyl-2-methyl-2-(4(trimethylsilyl)phenoxy)propionate (Silafibrate)is outlined in Figure 3.

^1^H-NMR (CDCl_3_) ^TM^ 7.44 (d, J = 8.45 Hz, 2 H), 6.93 (d, J = 8.45 Hz, 2 H), 4.15 (q, 2 H), 1.63 (s, 6 H), 1.27 (t, 3 H), 0.26 (s, 9 H)

*Animals*

Male Wistar rats (170–200 g) were used in this study. The animals were obtained from the animal house of Tabriz University of Medical Sciences and maintained under a controlled ambient temperature of 25 ± 2°C, with 40 ± 10% relative humidity and a 12 h light/12 h dark cycle. In each group, six animals were housed individually in polypropylene cages with paddy husk bed. They were fed with standard laboratory chow and tap water ad libitum. All animals were maintained and their care was conducted in accordance with the guidelines of the Tabriz University of Medical Sciences regarding the care and use of laboratory animals. 

*Pharmacological evaluation*

Clofibrate and its siliconized analog, silafibrate, were screened for their inhibitory effect on leukocyte accumulation in the air-pouch model of inflammation in rats. These experiments were carried out by observing the animals› response to carrageenan-induced inflammation, induced by injecting carrageenan into the air pouch. Both clofibrate and silafibrate were administered at doses of 2.5, 5, and 10 mg/kg through intragastric gavage.

*Anti-inflammatory activity*

Carrageenan-induced air-pouch model of inflammation was used to evaluate the anti-inflammatory activity of both clofibrate and the synthesized compound, silafibrate. Carrageenan-induced inflammation is used to assess the effectivity of nonsteroidal anti-inflammatory drugs (13). Air cavities were produced under light diethyl ether anesthesia by the subcutaneous injection of eight ml of sterile air into the intrascapular area of the dorsal part of the rats to open an oval-shaped space. Twenty-four hours later, four ml of 1% (w/v) carrageenan dissolved in saline was injected into the air pouch under light diethyl ether anesthesia. The carrageenan solution had been sterilized by autoclaving at 121°C for 15 min and supplemented with antibiotics (0.1 mg of penicillin-G potassium and 0.1 mg of dihydrostreptomycin sulfate per milliliter of the solution) after cooling to 35–40°C. Clofibrate and silafibrate were dissolved in polyethylene glycol 400.

The animals were divided into seven groups of seven rats each and, subsequently, vehicle, clofibrate (2.5, 5, and 10 mg/kg), and silafibrate (2.5, 5, and 10 mg/kg) were administered orally by gavages to the pouch-bearing rats one hour before injection of carrageenan, every 24 h, for six days. The final volume of the gavaged solutions was 0.2 mL per case. Six days after the injection of carrageenan solution, the rats were killed by cutting the carotid artery under deep diethyl ether anesthesia. The total pouch fluid was collected, and the volume was measured. The exudate samples were collected in glass tubes treated with ethylenediaminetetraacetic acid and refrigerated at 4°C until the time of counting. The pouch fluid was diluted 2-fold with saline, and the leukocytes in the fluid were enumerated using a hemocytometer. The granulation tissue that formed was also dissected and weighed. 

*Statistical analysis*

In this pharmacological study, the data are presented as mean value ± standard error of the mean (SEM). Statistical comparisons were made by the one-way analysis of variance (ANOVA) as appropriate. If the ANOVA analysis indicated significant differences, a Tukey’s post-hoc test was carried out to compare the mean values between the treatment groups and the control. Differences between the groups with a p < 0.05 were considered significant. Comparisons between the clofibrate and silafibrate groups receiving the same doses were carried out using the Fisher least-significant difference post-hoc test. In this test, differences between groups with a value < 0.05 were considered significant.

## Results

The inhibition of inflammation in the treatment groups receiving the same doses of clofibrate and silafibrate was evaluated with reference to the control group. The results are shown in Tables 1 and 2. Both compounds exerted inhibitory effect on leukocyte accumulation compared with the control group, but silafibrate was significantly more potent. The results of this study suggest that the replacement of Cl in clofibrate with the trimethylsilyl group increases the anti-inflammatory properties of the parent drug. 

## Discussion

Studies have indicated that siliconized compounds used as medicinal agents can retain or improve their biological profiles in comparison with those of their corresponding carbon isosteres (8, 11). In addition, the toxicity of siliconized isosteres of many drugs appears to be less than that of their carbon analogs (8, 11).

The silicon bioisostere shows interesting benefits in drug design. Sila-substitution at the 4-position of the piperidine ring of haloperidol significantly affects its pharmacological profile and metabolic fate in vitro (14). Replacement by silicon in a new chemical entity can lead to an improved pharmacological potency, modify its selectivity toward a given target, or change its metabolic rate (15). The sila-substitution can also increase the lipophilicity of a compound and hence increase its tissue distribution, particularly through membranes (including the blood–brain barrier), although with limitations of decreased water solubility (15).

## Conclusions

Silafibrate (Figure 1), a silicon analog of clofibrate (a PPAR-*α* agonist), was prepared from *p*-trimethylsilylphenol in a three-step synthesis. The results of the present study show that silafibrate has a potent anti-inflammatory effect compared to its parent compound, and this is similar to our previously reported results that siliconized aspirin had a greater inhibitory effect on carrageenan**–**induced edema than its parent compound.
